# Global sufferings, local voices: archival reactivations in Jewish theatre ephemera from Turkey

**DOI:** 10.1007/s10502-025-09514-9

**Published:** 2025-09-17

**Authors:** Rüstem Ertuğ Altınay

**Affiliations:** Interdisciplinary Laboratory for Performance and Politics, Department of Cultural and Environmental Heritage, University of Milan, Via Noto, 8, 20141 Milan, Italy

**Keywords:** Archive, Theatre, Ephemera, Jewish, Turkey, Holocaust

## Abstract

In Turkey, the principle of silence, *kayadez*, has long shaped Jewish cultural life and archival practices. While the community has remained cautious about maintaining archives and making them publicly accessible, some Jewish cultural producers have created unorthodox archival projects. Theatre has been an important realm for such efforts. Jewish theatre makers have often engaged with local and global Jewish archives in their works. More importantly, some of the ephemera associated with these productions were designed as archives in themselves. The souvenir book for *Anne Frank’ın Hatıra Defteri* [The Diary of Anne Frank] (dir. Albert Levi, 1958) presents a generative example of such projects. Bringing together a diverse array of historical and contemporary texts and images, from an excerpt from Frank’s diary to an acrostic poem about her, the book demonstrates how Jewish theatre makers have critically engaged with the globalized European archives of the Holocaust and produced new materials as they negotiated the politics of belonging in Turkey. In the face of historical trauma and antisemitism, the archive enabled alternative articulations of history, challenging the politics of memory in the present and enabling new visions and desires for the future. Jewish theatre makers in Turkey thus subtly articulated their own silenced yet painful experience of the Holocaust while imagining a global Jewish identity at a time when their communities and spaces had been rapidly disintegrating. Over the years, the meanings of this archival project have multiplied and shifted, offering new political promises, ambivalences, and challenges.

## Introduction

“Bro, do you actually read all this?” asked the young man who delivered another huge box filled with second-hand books and ephemera to my Istanbul apartment amidst the COVID-19 pandemic. Little did he know that he was not even the only person bringing dozens of these packages. Several stores on Nadirkitap, a comprehensive online database of second-hand booksellers in Turkey, worked with other delivery firms. No, of course I did not read all of them. “I need these for work,” I explained. I had recently launched a research project on minoritarian theatre practices in Turkey and its diasporas at a time when there was no live theatre. I had thus resorted to archival research not only to make up for the performances I could not attend but also to keep me busy during home confinement.

Most listings on Nadirkitap feature only the basic information about the books or documents, providing little description. Thanks to the limited interest in the materials I was collecting, which kept their prices affordable, I was able to follow a strategy of listing all items categorized under Theatre and ordering everything that I did not already have—except for the few original manuscripts that were beyond my budget. Following a principle of accidentality and an impossible desire for comprehensiveness, I thus built one of the largest theatre collections specializing in Turkey. Like all archival practice, my work depended on the invisible labor of blue-collar workers like that young man.

My rather unorthodox method of collecting, made possible by digital technologies and logistics corporations, facilitated my access to diverse materials, which I would not otherwise have sought or found. This is perhaps best demonstrated by the materials related to Jewish theatre in the Ottoman Empire and Turkey. The literature on the subject is extremely limited, and it provides few clues for collectors and archivists. A Nadirkitap search with the terms “Yahudi” [“Jewish”] and “oyun” [“play” or “game”] often yields only one result under the category of Theatre: Gotthold Ephraim Lessing’s 1749 play *Die Juden* [*The Jews*], translated into Turkish as *Yahudiler* (2000). The rest are mainly antisemitic conspiracy books (mostly by Islamist or Turkish ultranationalist authors) about the games Jews have supposedly played on Turkey and the world, such as *Siyonizmin Oyunları: Yahudinin Cihan Hakimiyeti Ülküsü* [The Games of Zionism: The Jew’s Ideal of World Dominance] (Anadol 1987); *Casusların Oyun Sahası Türkiye: İngiliz Yahudileri ve Dünyaya Yayılışları* [Turkey, the Playground of Spies: English Jews and Their Spread across the World] (Caba 2017); or *Son Casus: Yahudi’nin Tutmayan Oyunu* [The Last Spy: The Jew’s Failed Game] (Acar 2018).^1^ Even such a quick search attempt on the digital database of Nadirkitap thus reminds us how antisemitism has long prevailed in Turkey and how it can haunt research on Jewish cultural production. Despite such challenges, I was able to acquire diverse documents related to Jewish theatre, ranging from self-published plays to photographs and playbills.

Jewish people in Turkey are not simply a marginalized minority. They occupy a position of “national abjection” (Shimakawa 2002; Altınay et al. 2021; Çınar 2024). This precarious status defines not only the archival materials about the Jewish community but also their own archival practices. Given theatre’s significance as a site for negotiating the politics of national abjection, this is particularly true for theatre archives.

Julia Kristeva defines abjection as the process through which boundaries of the self are protected and (re)produced by jettisoning “what disturbs identity, system, order. What does not respect borders, positions, rules. The in-between, the ambiguous, the composite” (Kristeva 1982, p4). Performance theorist Karen Shimakawa adopts Kristeva’s psychoanalytical model as a lens to study the politics of nationalism, arguing that national abjects occupy “a role both necessary to and mutually constitutive of national subject formation” (Shimakawa 2002, p3). In this framework, the processes of nation-building involve not only the constitution of an “imagined community” (Anderson 1983), a collectivity constructed through the mediation of cultural production rather than immediate experiences and everyday encounters, but also constantly negotiating and maintaining the borders of this collectivity through abjection. These dynamics require national abjects to be both constantly made present and jettisoned (Shimakawa 2002). In the case of Turkey’s Jewish community, whose population is estimated to be 14,200 in a country of 85 million people as of 2023 (Jewish Virtual Library 2025), the conspiracy theories about crypto-Jews facilitate the operation of this tension.

The significance of memory for nation-building and the regulation of the politics of belonging entails a strong relationship between archives and national abjection. Karl Deutsch defines the nation as “a group of people united by a mistaken view about the past and a hatred of its neighbors” (Deutsch 1969, p3). The concept of national abjection reminds us how the loathing and fascination toward the groups “occupying the seemingly contradictory, yet functionally essential, position of constituent element and radical other” within the nation-state’s geographical borders is also a vital part of nationhood (Shimakawa 2002, p3). Archives contribute to these dynamics by facilitating the formation of national master narratives and the legitimization of nation-states (Berger 2013); the constitution of a national memory, consciousness, and identity (Brown and Davis-Brown 1998; Berger 1995); and the construction, reproduction, and re-imagination of geographical and cultural borders (Peña 2016). Public institutions, non-governmental organizations, private collectors, and other actors can thus explicitly or implicitly legitimize, reproduce, or challenge the categories of national abjection in and through their archival practices.

The political significance of archives endows them with possibilities for individuals who precariously negotiate the politics of national abjection. Of course, such promises are always limited by the distribution of resources as well as the very definition of the archive in the Foucaultian sense: “the law of what can be said,” the system “that establishes statements as events and things” and defines their enunciability and functioning (Foucault 1972, pp128–129). Nevertheless, minoritarian communities have long engaged in efforts to activate the powers of the archive. These efforts have involved critical engagements with materials in mainstream archives—which, otherwise, are often sites of silencing if not violence for them—as well as the creation of alternative archival projects of various scales and forms. Such projects can best be described as “archives of abjection” (Altınay 2024a, p13).

In and through archives of abjection, minoritarian communities explore the historical processes through which they have come to occupy positions of abjection and develop alternative narratives about the past. Such archival practices offer them opportunities to question the politics of belonging in the present and imagine the possibility of alternative futures. It is worth noting, however, that these archival practices are not necessarily characterized by an ultimate resistance to hegemonic power relations or an uncompromising investment in liberatory or progressive politics. They often involve difficult negotiations, political ambivalences, and erasure or violence targeting other minorities—including those occupying comparable positions of national abjection. In the context of Turkish theatre, for instance, queer politician, medical doctor, historian, memoirist, playwright, librettist, and dramatic translator Rıza Nur propagates ultranationalist, racist, misogynist, and eugenicist views as he negotiates the sexual politics of belonging in and through his exilic archival practice, which involves his dramatic texts alongside a diverse array of other materials (Altınay 2021). The Jewish feminist playwright, theatre critic, and historian Beki L. Bahar, on the other hand, exercises historical erasure in her engagement with Jewish and Ottoman archives to claim that Jewish boys were not involved in the Ottoman Empire’s popular queer performance cultures (Bahar 2007; Çınar 2024). To prevent members of her community from an intersectional abjection as an ethnoreligious and sexual minority, Bahar thus reproduces the heterosexism that dominated mainstream theatre historiography in Turkey for decades (Altınay 2024b). Similarly, the explorations for identifications and forms of belonging beyond the nation-state in Jewish theatre archives do not simply challenge the nation-state and the exclusions and violence it involves but also risk feeding into Zionist discourses and political projects that present Israel as the (only) homeland for Jews and legitimizes the occupation of Palestine and settler colonialism. The souvenir program book for the Türk-Musevi Sahne Amatörleri’s [Turkish Jewish Stage Amateurs] 1958 production *Anne Frank’ın Hatıra Defteri* [*The Diary of Anne Frank*] (dir. Albert Levi), which I acquired through Nadirkitap, presents an excellent example to such tensions.

*Anne Frank’ın Hatıra Defteri* was based on *The Diary of a Young Girl* by the Holocaust victim Anne Frank (1929–1945), a text that played a fundamental role in the creation of a transnational memory of the Holocaust (Assman 2010). The play was produced by the Galata, Beyoğlu, Kasımpaşa, Şişli Musevi Cemaati Yardım Kolu [Galata, Beyoğlu, Kasımpaşa, Şişli Jewish Community Charity Branch], today the Neve Şalom Sefarad Sinagogları Vakfı [Neve Shalom Sephardi Synagogues Foundation]. It is noteworthy, however, that theatre makers at Arkadaşlık Yurdu Derneği [Friendship Home Association], which included the director Levi and the translator Filon Koen, had already staged an adaptation of *The Diary of a Young Girl* in 1948 (Bihar 2011). Like other Jewish community organizations in Turkey around that time, the association’s productions focused primarily on Jewish mythology and history, such as *Yusuf ile Züleyha* [Joseph and Zuleikha] (1947) and *Alfred Dreyfus* (1947). Over the years, they continued their Holocaust-themed productions with plays such as *Rambam Hastanesi* [Rambam Hospital] (1968–1969) (Bihar 2011).

While most of the Holocaust-themed plays from the mid-twentieth century have been lost and forgotten, productions based on *The Diary of a Young Girl* have retained their popularity among Jewish community theatres as well as mainstream private and public theatre companies in Turkey to this day. In fact, only one year before the Turkish Jewish Stage Amateurs, the Ankara State Theatre had staged *Anne Frank’ın Hatıra Defteri* (dir. Cüneyt Gökçer, 1957), which had toured in Istanbul as well (Korucu 2016). At a time when Turkey enjoyed friendly relations with Israel, this highly successful production also sparked broader public debates regarding the Holocaust and inspired other events, such as a panel dedicated to the play. Organized by Sanat Sevenler Kulübü [Art Lovers Club] in Ankara, the panel featured academics and politicians such as Turan Güneş, Bahri Savcı, and Bedrettin Tuncer and involved discussions on human rights (Anne Frank 1957). The popularity of the Ankara State Theatre production may have inspired and encouraged the amateur Jewish theatre makers to stage the play again. In that regard, this choice may have been a strategy to reach out to what the community calls *geniş toplum* [the broad society], the predominantly Muslim population in Turkey, and foster their sympathy for Jews as well. Given that the Ankara State Theatre production did not feature any Jewish theatre makers, the Turkish Jewish Stage Amateurs production would immediately make the connection between the stage performance and Turkey’s Jewish community. As such, this production was also an attempt by the community to tell what they regarded as their own story.

Staged at the Tepebaşı Dram Tiyatrosu [Tepebaşı Drama Theatre], a venue owned by Turkey’s most established public theatre company İstanbul Şehir Tiyatrosu [Istanbul City Theater], *Anne Frank’ın Hatıra Defteri* served community-building and fundraising among the Jews of Istanbul. The producers enhanced their fundraising efforts with *Anne Frank Temsilinin Hatırası* [The Souvenir for the Play Anne Frank], a souvenir program book sold separately to the audiences (G. B. K. Ş. Musevi Cemaati Yardım Kolu 1958). What made this book exceptional is its archival nature, in part grounded in the production’s historical and documentary content. *Anne Frank Temsilinin Hatırası* brings together a welcome note introducing the activities of the charity, a poem about helping the poor and the sick, advertisements for the Jewish-owned cosmetics brand PE-RE-JA, a cast list and their photographs, newspaper articles about Frank, an excerpt from Frank’s diary and its (mis)translation into Turkish, an acrostic poem about Frank by the translator Filon Koen, and an apology note addressed to Frank by the director Albert Levi. This rich and diverse collection is particularly meaningful in face of the “archival turn,” a shift from the treatment of the archive as source to the archive as subject, most closely associated with the work of Michel Foucault (1972) and Jacques Derrida (1996) (Stoler 2002).

With the archival turn, the archive began to “serve as a strong *metaphor* for any corpus of selective forgettings and collections—and, as importantly, for the seductions and longings that such quests for, and accumulations of, the primary, originary, and untouched entail” (Stoler 2002, p94). The expanding definitions of the archive have concerned professionally conservative archivists like the former president of the Society of American Archivists William J. Maher, who frames the “misuses” of the term to refer to personal collections or electronic databases as “an attempt by the scholar or database builder to lend panache or cachet and an air of respectability to what otherwise might be little more than a personal hobby or collecting fetish” (1998, p254). Given the intensely personal, fetishistic, and ephemeral forms archives of abjection can take, Maher’s accusations actually suggest how the archival turn can provide us with the theoretical and methodological tools for studying the politically significant yet unorthodox forms of archival practice, as *Anne Frank Temsilinin Hatırası* exemplifies.

As an archive of abjection, the souvenir program book demonstrates how members of Turkey’s Jewish minority have critically engaged with the globalized European archives of the Holocaust and produced new materials as they negotiated the politics of belonging at a time when their communities and spaces had been rapidly disintegrating. With these diverse materials, as they mourned the passing of Frank and processed the trauma of the Holocaust, they also challenged *kayadez*, the strategy of silence defining the everyday experiences of the community. The Jewish community of Istanbul thus subtly articulated their own silenced yet painful experience of the Holocaust and antisemitic violence in Turkey while also imagining a global Jewish identity. The dimension of charity in the archive and the live performance facilitated a politics of solidarity among members of Turkey’s diminished Jewish minority and suggested the possibility of a future for the community against all adversity. Romanticizing the politics of resistance in this archive of abjection, however, would risk ignoring the complex matrices of power within which the archive gains meaning. Treating resistance as “a diagnostic of power” (Abu-Lughod 1990, p41), instead, reveals how *Anne Frank Temsilinin Hatırası* as an archive of abjection is now also vulnerable to the contemporary critiques of the memory work associated with the Holocaust and a globalized pogromatic discourse facilitating Zionism and the colonialist policies of Israel as well as the United States (Shohat 2017; Hepkaner 2018; Finkelstein 2000).

Projects like *Anne Frank Temsilinin Hatırası* paved the way to diverse, at times much larger efforts combining archival practice and performance, such as the quincentennial commemorations for the arrival of Sephardic Jews in the Ottoman Empire following their expulsion from Spain and Portugal in 1492. In and through such projects, Turkey’s Jewish community have created and reactivated archives through which they both reproduced and challenged hegemonic historical narratives defining their experiences in terms of Ottoman and Turkish tolerance and saviorhood and Jewish gratitude, intervened in national and global politics, and explored new visions for the future.

## Jewish theatre archives in the face of kayadez

Any investigation into the Jewish politics of memory, archival practice, and cultural production in Turkey must acknowledge the foundational concept of *kayadez*—which, according to the Turkish Jewish political scientist Karel Valansi, “can summarize all the Jewish experience in Turkey” (2018, p120). Kayadez or kayades is a Judeo-Spanish word that literally translates as “silence” or “keeping silent” but “[in] reality it means; don’t be vocal, don’t criticize, don’t oppose, stay silent,” urging “Turkish Jews to be hidden and almost be invisible in the public sphere” (Valansi 2018, pp120-121). As such, kayadez is not so much a viewpoint as it is a way of life (Aviv 2021, p8). Although the scholarly and public debates tend to frame kayadez as an essentially Turkish phenomenon, it was a common strategy in all pre-modern diasporic Jewish societies, including those in the Ottoman Empire (Rodrigue 2020). A major reason for the misinterpretation of kayadez as an exclusively or primarily Turkish phenomenon is its continuing significance in contemporary Turkey as opposed to several other contexts where Jewish lives are not marked by similarly precarious negotiations of the politics of belonging anymore.

In the republican history of Turkey, traumatic events like the Elza Niyego (or Niego) affair have fostered kayadez. Niyego was a young Jewish woman who was murdered in 1927 by a Muslim Turkish official whose advances she had rejected. The murder sparked an antigovernment demonstration at her funeral that was regarded as criminal, resulting in the arrests of several Jewish protestors. Moreover, the press accused Jews of agitation against the state and their freedom of movement was restricted (Akturk 2009). The Niyego affair suggests how the media as well as the law have contributed to the consolidation of kayadez by reinforcing silence.

The way kayadez regulates Jewish people’s relationship with the state in Turkey is best summarized by a Judeo-Spanish motto featuring Turkish words: “No moz karışayamoz a la eços del hükümet” [We do not interfere in the government’s work] (Diler 2015). Developed through many traumatic encounters with the state, this saying reminds members of Turkey’s Jewish minority to avoid getting involved in politics (Aviv 2021). Theatre scholar Verda Habif, also a member of the community, argues that “taking a ‘silent’ attitude is not just a survival strategy, but it also indicates a deep mistrust of both the government and the media” (2021, p83). I would add to Habif’s list a mistrust of the broad society as well. The dynamics of kayadez can become so complicated that silencing and self-silencing are often indiscernible, especially in the archive. Hence, when anthropologist Marcy Brink-Danan (2012) discovers that the pages of newspapers documenting the 1986 Neve Shalom massacre have been taken out of the newspaper collections in Turkey’s National Press Archives, she considers a Jew trying to protect their community as likely a culprit as any other.

Kayadez reflects the experiences and founding myths that define the Jewish community in the Ottoman Empire and Turkey. Jewish presence in Asia Minor dates to the fourth century BCE and the contemporary Jewish community in Turkey is the amalgamation of diverse populations who have migrated to the Ottoman Empire and Turkey over the centuries (Brink-Danan 2012). The arrival of Sephardic Jews in the Ottoman Empire following their exodus from Spain and Portugal in 1492 and subsequent waves of migration resulted in a relative homogenization. Thanks to their demographic and economic power, Sephardic Jews dominated the cultural, religious, and linguistic life of Ottoman Jewry, sometimes at the expense of the local Jewish traditions (Şişman 2015).

The Sephardization of Jewish life has shaped the politics of historiography and archival practice as well. Turkey’s official historiographical narrative and many public accounts of the members of Jewish community mark 1492 as the starting date of Jewish presence in Asia Minor (e.g. Erol 1992). This historiographical framework frames all Jewish life in Turkey with reference to a founding antisemitic trauma that is located outside of the country. Even more importantly, it positions the Muslim Ottomans and Turks as tolerant hosts while burdening Jews with an eternal debt (Brink-Danan 2012; Baer 2020). This historiographical framework gained significance in the aftermath of the Ottoman Reform Edict of 1856, which promised equality in education, government appointments, and administration of justice to all religious communities, termed *millet*. As the Jews engaged in competition with other non-Muslim communities for public resources, they employed a discourse of gratitude to prove their loyalty to the Ottoman Empire (Cohen 2020). As part of these efforts, the community invented the quadricentennial commemoration for the Sephardic Exodus to the Ottoman Empire as a patriotic holiday (Cohen 2020). Toward the end of the nineteenth century, the discourse of loyalty overlapped with the category of the “model *millet*, a group that Ottoman intellectuals and politicians could conjure in order to think through issues of Ottoman modernity, tolerance, and imperial citizenship” (Cohen 2020, p222). The performance of loyalty and model minority fostered kayadez as well.

The discourses of Ottoman tolerance and saviorhood and Jewish gratitude, and the archives sustaining them, continued to be mobilized by different actors at critical times in history, such as the quincentenary of the Jewish Exodus, commemorated for three years in 1990–1992. This time, the celebrations also presented Turkey as a savior of Jews during the Holocaust on the grounds that the country received a small number of refugees, most notably academics, and some Turkish diplomats took action to help Jewish people escape the Holocaust. The commemorations, sponsored by the Turkish government, the Jewish community of Turkey and members of the American Sephardi Foundation, built on this historiographical framework in their efforts to improve the country’s image at a critical time when Turkey integrated into the global market economy (Bali 2009; Hepkaner 2019; Baer 2020). The events particularly aimed to erase the memory of the violent coup d’état of 1980; prevent the recognition of the Armenian Genocide; silence the demands of the Kurdish ethnic minority; empower Turkey in the Cyprus conflict; create awareness about the rights violations targeting Turkish minorities in other countries, especially in Bulgaria; warm Turkey’s diplomatic relations with Israel; and obtain international support for Turkey’s candidacy for European Union membership (Bali 2009). While the affectively charged historiographical framework of tolerance empowered some elite Jews and enabled them to become powerbrokers, it also framed Jews as forever guests in “Turkish lands” who can never be full members of the nation. Neither objects nor subjects, the Jewish abject facilitates the production of a national identity and consciousness as well as the processes of national subject formation through its cultural, symbolic, and at times legal exclusion (Shimakawa 2002).

The discourse of ethnoreligious tolerance hides a history of atrocities against Jews in the Ottoman Empire and Turkey, including discrimination, violence, and forced conversions in the Empire, the rights violations and silencing in Turkey’s formative years as in the Elza Niyego affair, the monolingualism campaigns and policies of the 1930s, the Thrace Pogroms of 1934, Turkey’s antisemitic policies during the World War II, discriminatory tax policies—most notably the Varlık Vergisi [Wealth Tax] of 1942, which facilitated the transfer of wealth to create a Muslim bourgeoisie at the expense of the established non-Muslim one—and the Istanbul Pogrom of 1955 as well as the present-day antisemitism in the country (Bali 2009; Şişman 2015; Baer 2020).^2^ It is in response to these histories of violence that kayadez has gained prominence as a survival strategy.

Over the years, Turkey’s Jewish community’s need for silence and invisibility might have intensified with the shrinking of their population following migrations to Israel, Europe, and the Americas; the disappearance of Jewish neighborhoods and institutions; the ramifications of the Israeli-Palestinian conflict on their lives; the rise of political Islam in Turkish politics and its propagation of antisemitic discourses while maintaining close military and commercial relations with Israel (as most recently seen during the 2023 Israel–Hamas War); and the popularity of antisemitic conspiracy theories across the political spectrum in the post-truth age. Regardless of its power, however, kayadez does not entail absolute self-censorship and silence; it is also productive in complex ways that shape the performance of everyday life and archival practice. In language, kayadez manifests in lexical choice and the frequent use of parables and practical jokes (Aviv 2021; Demirel 2017). It also shapes the production of knowledge accessible to the broad society, especially by Jewish media professionals (Aviv 2021). These concerns have shaped Jewish archives as well.

In archival practice, kayadez informs the principle of “papeliko sataniko,” meaning “small bits of paper are little demons” (Bali 2020). The Judeo-Spanish saying warns members of the community that documents can unexpectedly disappear and show up in unlikely places and create trouble (Bali 2020). According to the Turkish Jewish popular historian Rifat N. Bali, in the 1970s, the saying primarily referred to illegal commercial transactions, often conducted to avoid customs or taxes (Bali 2020). Over time, its meaning expanded to cover all written records. The Jewish community thus destroyed many of its communal archives and censored others (Brink-Danan 2012; Bali 2020). An emblematic case of archival destruction is the case of the Zionist youth who conducted their activities secretly. When they migrated to Israel following the country’s declaration of independence in 1948, they destroyed their archives to protect those who stayed in Turkey (Bali 2020).

Kayadez manifests in the form of centralized instances of censorship as well, as the exhibitions organized at the Quincentennial Foundation Museum of Turkish Jews demonstrate. The Museum is the community’s official memory space accessible to the broad society. There have been instances when the museum officials refused to feature the traumatic events in the history of the community in their exhibitions to maintain the discourse of tolerance and the positive tone they took pride in (Brink-Danan 2012). Researchers can access some official archives in Turkey, such as the Izmir Jewish Community Foundation archive (Yılmaz 2023). Others, like the Chief Rabbinate Archive, are not open to researchers (Gülşen 2021). The Chief Rabbinate of Turkey Foundation has donated some of their collections, possibly those that pose fewer risks to community members, to eminent US universities (Hepkaner 2020). With this strategy, they have made selected resources available to elite international researchers and enhanced their value and respectability, thereby amplifying their capacity to produce a truth-effect while limiting the access of the broad society in Turkey as well as of the diasporic descendants of Ottoman and Turkish Jews to their archives.

The Jewish community’s caution about archives might have informed their investment in what Diana Taylor (2003) diagnoses as the repertoire: the embodied transfer of social memory through performance. In the parts of the Ottoman Empire that would later host the Republic of Turkey, the archival materials documenting Jewish involvement in multicultural theatre activities date to the early thirteenth century (And 1969). Following the migrations from Thessaloniki and the Iberian Peninsula in the fifteenth century, Sephardic Jewish performers staged physical performances such as juggling and acrobatics (Bahar 2007). Over the years, Jewish performers played a significant role in the development of popular Ottoman performance genres, especially the shadow theatre *Karagöz* and *orta oyunu* (theatre-in-the-round), an improvisational comedy genre that shares stylistic elements and, according to some researchers, historical connections with commedia dell’arte (And 1969; Bahar 2007; Hepkaner 2018).

European-style theatre began to gain unprecedented popularity in the Ottoman Empire with the Europeanization and modernization reforms implemented during the *Tanzimat* [Reorganization] Period (1839–1876) (Adak and Altınay 2018). Christian communities, especially Armenians, played a central role in the development of Ottoman and Turkish theatre. Despite their earlier work, the contributions of the Jewish community to the development of European-style theatre remained limited. The reasons, according to Beki L. Bahar (2007), included the clergy’s anti-theatrical pressure and their prohibitive attitudes regarding women’s stage presence. The early performances included productions by Jewish charity organizations, some private companies, and school plays in French and Judeo-Spanish, performed at the homes of community members and at Armenian venues (Bahar 2007). In this period, under the influence of the French-language schools for Jewish children established by the Alliance Israélite Universelle, theatre’s pedagogical function was central (Borovaya 2012). Theatre’s ability to address illiterate segments of the population and its facilitation of the embodied transfer of social memory and language were also important in that regard.

Following the Young Turk Revolution (1908), while the association of Jews with the Imperial “center” persisted during the Second Constitutional Era (1908–1923), the meanings of this association multiplied (Cohen 2020). The discourses and practices regarding the model minority status of Jews coexisted with the antisemitic conspiracy theories and concerns proliferating amid intensifying debates about Zionism and the supposed role Jews played as the masterminds behind the revolution (Cohen 2020). In this period, Jewish theatre emerged as a political propaganda tool for Zionism (Borovaya 2012). These tensions that defined Jewish life heightened with the transition from the Ottoman Empire to the Republic of Turkey. In the early years of the nation-state, following its inception in 1923, the Jewish community’s generally positive relations with the state elite existed in tension with antisemitism in the media and among the broad society, in part fueled by the rise of antisemitism in Europe (Levi 1992). In 1925, the community responded to these dynamics by renouncing the rights granted to them with the Treaty of Lausanne as a religious minority (Levi 1992; Gülşen 2021). This strategy, however, did not ensure the safety of the community. A traumatic breaking point was the Thrace Pogroms of 1934, conducted by members of the local state elite, officials from the Republican People’s Party in government under a single-party regime, and antisemitic Turkish ultra-nationalists (Eligür 2017). Theatre is a critical site where minoritarian subjects negotiate the politics of national abjection and this was particularly true for the Jewish community in Turkey (Shimakawa 2002; Altınay et al. 2021; Çınar 2024). In the aftermath of the pogroms, some Jewish playwrights, such as Yakim Bahar (1935), created works in Turkish where they explored the possibility of self-Turkification and attained mainstream success with public theatre productions. Otherwise, school and community productions continued to serve as the main venue for Jewish theatre. An important aspect of these works is their limited accessibility to the broad society, and thereby their compatibility with kayadez. After the 1930s, while the Turkification policies resulted in Turkish becoming the main language of the productions, the venues largely remained the same (Bihar 2011). Over the years, as the community became alienated from Judeo-Spanish, most Jewish theatre makers gradually employed Turkish as the main language of the plays and limited the use of Judeo-Spanish to the creation of comedic or nostalgic effects in plays that are otherwise entirely in Turkish (Bihar 2011). Today, events like the Ladino Day feature plays in Judeo-Spanish as part of the efforts to keep the language alive.

The availability of archival materials related to Jewish theatre in Turkey is extremely limited, and kayadez is not the only reason. To begin with, the very possibility of archiving performance is a contested matter in the literature (Phelan 1993; Taylor 2003). Such concerns are present not only in performance theory but also in everyday archival practice, exacerbated by the structural issues in Turkish academia. In Turkey, theatre departments focus on studio training and faculty mostly comprises teaching artists (Adak and Altınay 2018). The limited scholarship on theatre is largely produced in Turkish language and literature, history, and the few dramaturgy and cultural studies departments. Reflecting the dominant theoretical and methodological conventions in these fields, this literature overwhelmingly focuses on dramatic texts at the expense of ignoring other aspects of theatre production. It also reproduces the text/performance divide and the hierarchically conceptualized relationship between text and performance (Meisner and Mounsef 2011). This conceptualization has faced major challenges, especially with the rise of performance studies and the performative turn in the humanities since the 1980s (Fischer-Lichte 1999; Diamond 1996). Nevertheless, the notion of textual authority has retained its explicit or implicit significance in many contexts, including Turkey. Limiting research to textual analysis can also facilitate the production of research outputs by reducing the need for archival and ethnographic research. This is important in view of the lack of funding in the humanities and the neoliberal demand for productivity, which are growing problems in Turkey (Altınay et al. 2021). In the case of teaching artists, such concerns often intersect with the market demands, further marginalizing research on minoritarian theatre archives, especially in amateur contexts. Under these circumstances, theatre collections at most public repositories are limited to dramatic texts, rendering ephemera difficult to access.

Turkey’s only specialized theatre library, the Refik Ahmet Sevengil Theatre Library, opened in 2011 as part of the Turkish State Theatres. Since the collection comprises primarily documents about the history of the Turkish State Theatres, founded in 1949, the materials on Jewish theatre makers are extremely limited. Some special collections at other public repositories feature materials related to the few Jewish playwrights who attained mainstream success in Turkey. A recent and important example is the Sevim Burak Archive at the Mimar Sinan Fine Arts University Library, acquired in 2022. I must note, however, that the archive was promoted with reference to Burak’s career as a pioneering postmodern novelist and short story writer rather than her plays—and this was probably the primary reason why the university acquired them. The absence of Jewish theatre archives becomes even more visible in view of the emerging collections related to other minoritarian theatre and performance archives, such as the Armenian periodical *Kulis* [Backstage], recently digitized by the Hrant Dink foundation as part of their Hagop Ayvaz Collection. Nevertheless, some Jewish performers also find their ways into other digital archiving projects, such as the Reşad Ekrem Koçu and *İstanbul Ansiklopedisi* [Encyclopedia of Istanbul] Archive at SALT Research, featuring the work of the eminent queer popular historian.

An important collection for Jewish community theatre was in the Chief Rabbinate of Turkey Foundation archives, which they have donated to the Harvard College Library’s Judaica Collection (Hepkaner 2020). Another significant archival site for theatre materials, especially ephemera, has been the Jewish neighborhood associations (Hepkaner 2020). With the shrinking population, several of these associations closed down or merged with others. They often lacked the resources to maintain an organized archive and, as I know from the interviews I conducted with community members and theatre makers in 2022–2023, some archival materials were lost as the associations moved to new spaces, and members took others to their homes. As people passed away or migrated, second-hand booksellers acquired some of these materials. This is probably how *Anne Frank Temsilinin Hatırası* found its way to my archive by way of the Nadirkitap website.

## Surrogating Annecke, remembering the self

In performance studies, following Diana Taylor’s (2003) distinction between the archive and the repertoire—which is too often interpreted to be stricter than Taylor intends—theatre is more immediately associated with the embodied transfer of social memory. Theatre practices, however, both reactivate archives and produce diverse archival materials, some of which are designed as archives in themselves. Such endeavors are particularly important for archives of abjection, as the souvenir program book *Anne Frank Temsilinin Hatırası* demonstrates.

*Anne Frank’ın Hatıra Defteri*, staged in April 1958, was a community performance produced for charity purposes in the aftermath of traumatic events for Turkey’s Jewish community on multiple and connected scales. The Thrace Pogroms of 1934 erased the once strong Jewish presence from the region and fueled self-assimilation debates among Turkey’s Jewish communities. During World War II, Turkey was the only neutral country to implement anti-Jewish laws. The country denaturalized 3,000 to 5,000 Jews living abroad while between 2200 and 2500 Turkish Jews were deported to Nazi extermination camps (Baer 2020). In 1941, Istanbul was the site of the *Struma* disaster, where the engine of a ship carrying Jewish refugees from Romania to the British Mandate of Palestine failed in the Bosphorus. With the pressure from Germany and the United Kingdom, and the existing antisemitic laws that banned Jews persecuted in their countries of nationality from entering or residing in Turkey, the Turkish authorities did not permit the passengers to disembark. A Soviet Navy submarine torpedoed the *Struma*, killing more than 750 refugees and members of the crew. Also in 1941, non-Muslim men between the ages of 27 and 40 were conscripted into the army to perform physical labor, regardless of whether they had performed the compulsory military service or not. In 1942, the Wealth Tax aggravated the financial burden of the conscription on Jewish and other non-Muslim families, resulting in many Jewish businesses and real estate changing hands. The men who could not pay the tax were sent to labor camps. Only three years before the production, the Istanbul Pogrom of 1955, targeting Istanbul’s Rum (i.e., Greek-speaking Orthodox Christian) minority affected many Jewish-owned businesses and homes as well. Even more importantly, the Istanbul Pogrom intensified the sense of precarity that haunted Jewish life in Turkey. Under these circumstances, many Jews migrated from Turkey to Israel as well as to Europe and the Americas. The producers and audiences of *Anne Frank’ın Hatıra Defteri* had witnessed many if not all these events. Nevertheless, this was also a time when Turkey and Israel enjoyed close relations. Turkey was the first country with a predominantly Muslim population to recognize Israel, and they were allies throughout the 1950s. The Ankara State Theatre’s 1957 production of the play—directed by pro-government Cüneyt Gökçer, who would become the Artistic Director of the State Theatres in 1958—also reflected this environment. The Turkish Jewish Stage Amateurs responded to these tensions.

The production and the souvenir program book served as a site of melancholia for Turkey’s Jewish community in the aftermath of the loss of Jewish lives, both literally and figuratively. José Esteban Muñoz argues that for minoritarian subjects, “melancholia [is] not a pathology or self-absorbed mood that inhibits activism, but... a mechanism that helps us (re)construct identity and take our dead to the various battles we must wage in their names and in our names” (1999, p74). In this framework, archives of melancholia supply “a necessary history to a collective struggle” (Muñoz 1999, p74). The internationally popular diary of a Jewish girl from Germany who died at the Bergen-Belsen concentration camp at the age of 16 emerged as a politically safe and affectively powerful archive of melancholia in the face of kayadez in Turkey. Moreover, thanks to the Ankara State Theatre production, it was now an archive approved by the state. Through the production and the souvenir book, the Jewish community processed historical traumas and responded to the ongoing antisemitism in the country. In a way that reflects the productive dimension of kayadez, the diverse documents in the souvenir book serve this amalgamation of political activism and melancholia in complex ways.

**Fig. 1:**
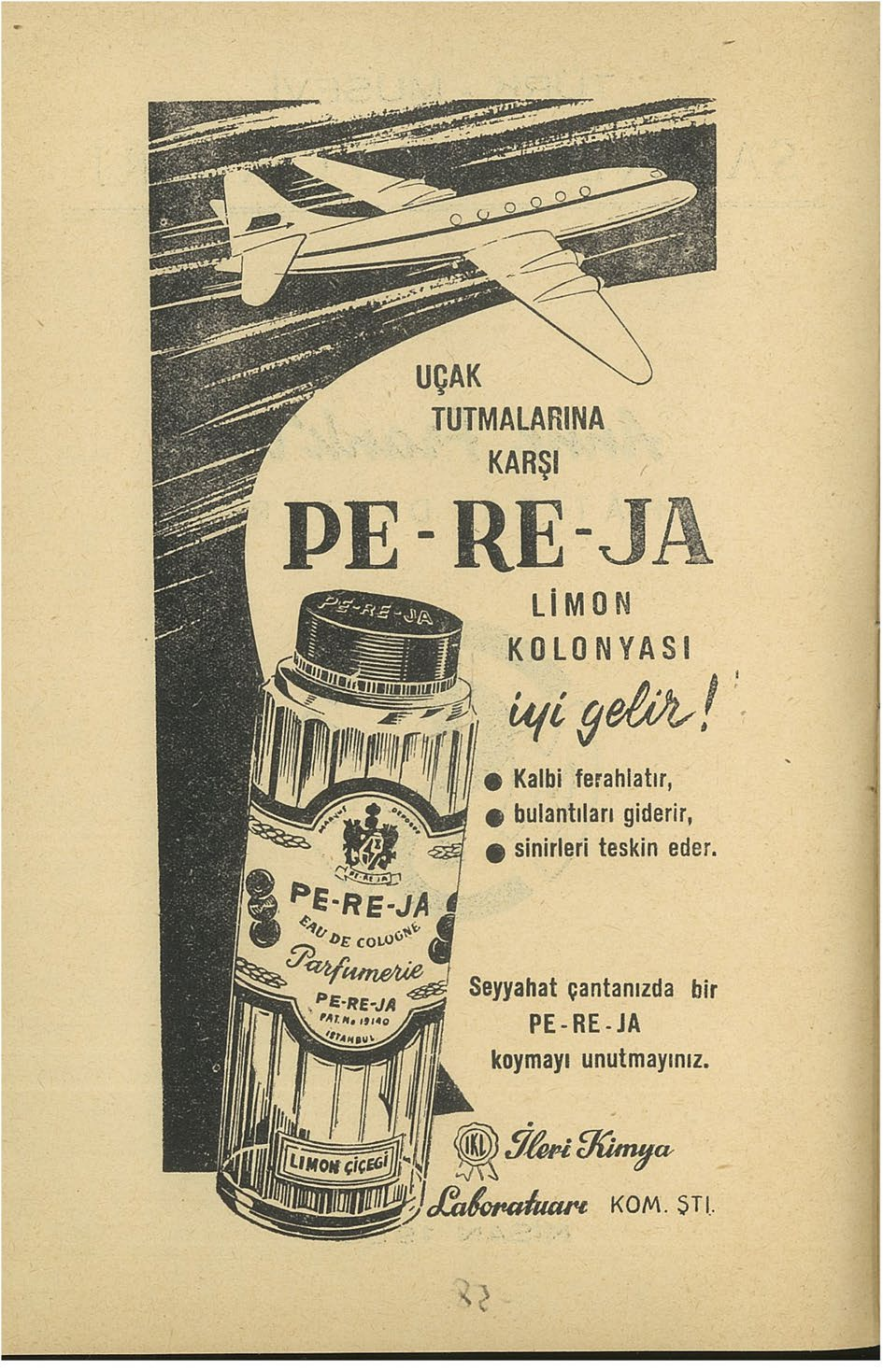
Air travel-themed advertisement for PE-RE-JA (G. B. K. Ş. Musevi Cemaati Yardım Kolu 1958, p4)

Following the inner cover, featuring the logo of Turkish Jewish Stage Amateurs, the title of the play, and the date, the first material in the book is an advertisement for the lemon-scented cologne produced by the successful Turkish Jewish cosmetics brand PE-RE-JA, which appears to have sponsored the publication. In Turkey, lemon-scented cologne is a popular product used for refreshing and sanitizing purposes. The advertisement, featuring the drawing of an airplane and a cologne bottle, asserts that the PE-RE-JA lemon cologne is good for airsickness as it “refreshes, ends nausea, and calms down the nerves” (Fig. 1). In 1958, air travel was less common, more expensive, and indeed less comfortable than it is today. It was also a necessity for many Jews in Turkey, many of whom contemplated emigration or whose families and friends were now internationally dispersed. With its emphasis on airsickness, the advertisement implicitly acknowledged the challenges involved in migration and in remaining in physical contact with loved ones. This targeted advertisement, in a publication reflecting solidarity between Jewish entrepreneurs, charity organizations, and theatre makers, encouraged members of the community to support Jewish businesses as well. Even more importantly, it reassured audiences that it was still possible for Jews to own businesses in Turkey. The fact that the other four advertisements in the book are also for PE-RE-JA, however, implies the devastating economic transformations the community endured.

**Fig. 2:**
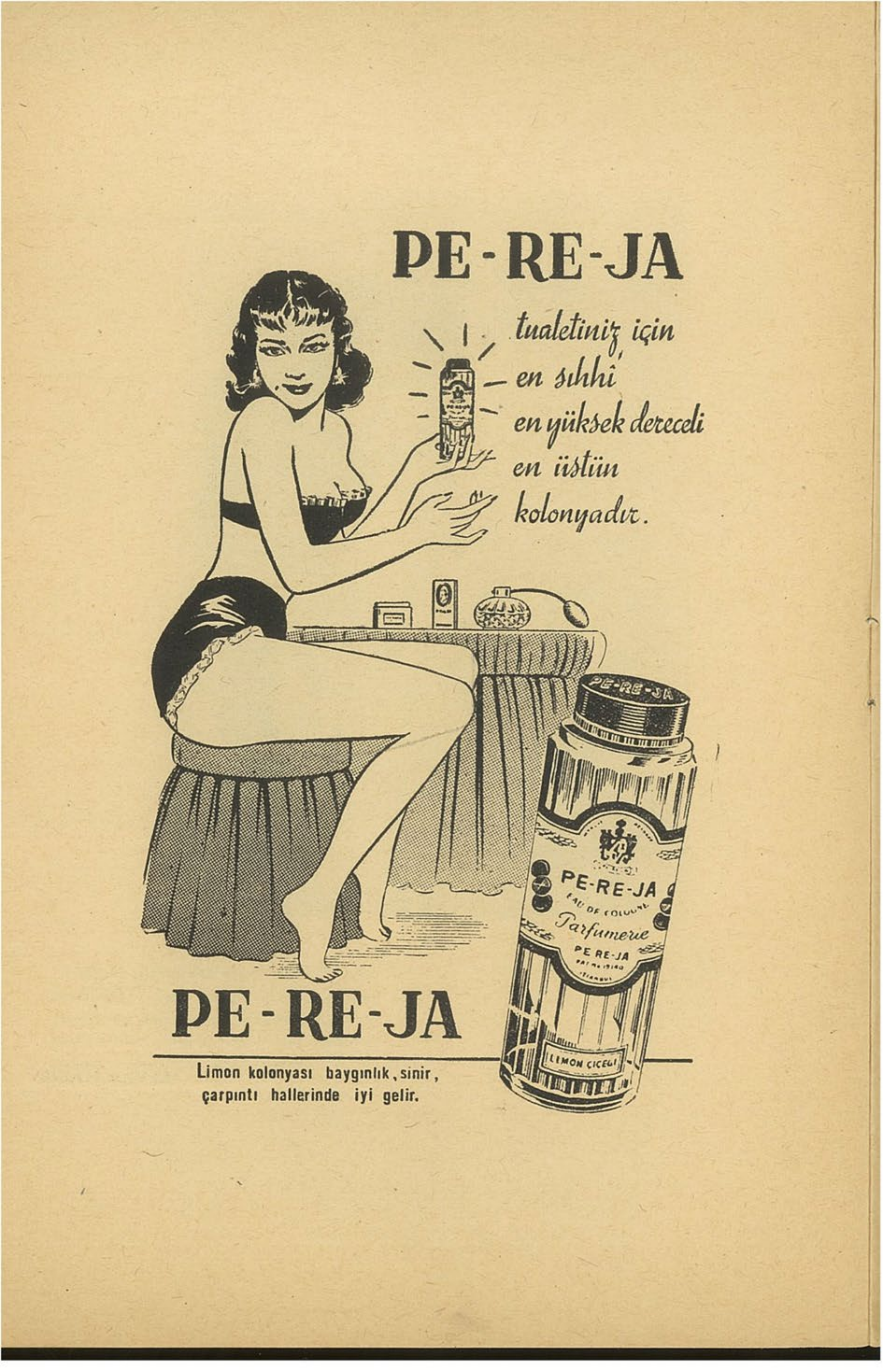
An erotic advertisement for PE-RE-JA (G. B. K. Ş. Musevi Cemaati Yardım Kolu 1958, p8)

The other three PE-RE-JA advertisements in the book reflect the rise of eroticism in the Turkish advertisement industry during the Cold War. Two of these illustrations depict women in lingerie at dressing tables while the third one shows a woman wearing a towel in a modern bathroom, enjoying the cologne (Fig. 2). The gendered and sexualized consumerism in the advertisement, which is in stark contrast with the tragedy of Anne Frank—a Jewish girl who could never grow up—reassures readers and audiences that regardless of how vivid its memory might be, the Holocaust is over. In that regard, one of the advertisements being published side-by-side with the director Albert Levi’s apology note to Frank is striking. Titled “Annecke,” Levi’s note reads “We tried to bring your memories to life in order to honor your soul….. Don’t be upset with us, alright…..?” (G. B. K. Ş. Musevi Cemaati Yardım Kolu 1958, p9). This brief note performs an intense hesitancy with its semantic content as well as its unconventional use of punctuation. Community theatre is often a site where participants experience and process shame or embarrassment (Kunin 2019). In a post-Holocaust Jewish performance like *Anne Frank’ın Hatıra Defteri*, these challenging affective processes become entangled with survivor’s guilt. The publication of this melancholic apology note next to the erotic advertisement praising PE-RE-JA as “the most sanitary, the highest degree, the greatest cologne for cosmetics” (Fig. 2) also serves as an acknowledgment of (and perhaps a call for) Jewish life in the aftermath of trauma.

**Fig. 3:**
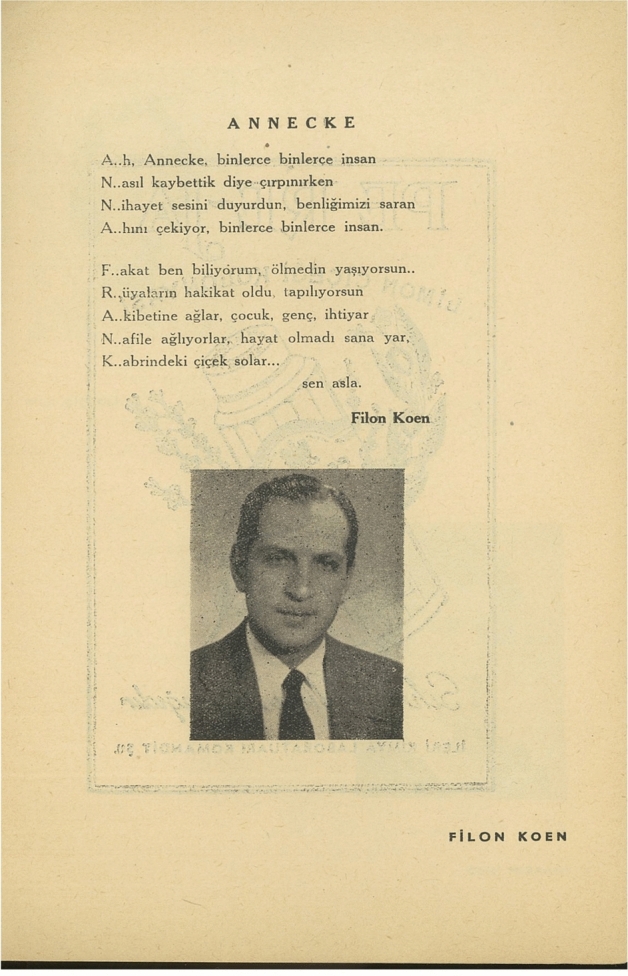
Filon Koen’s acrostic poem for Frank (G. B. K. Ş. Musevi Cemaati Yardım Kolu 1958, p8)

The Turkish Jewish survivor’s guilt and the melancholic attachment to Anne Frank are present in the acrostic poem “Annecke” by the translator Filon Koen, who also played Otto Frank in the production, as well. Acrostic poetry has a childlike and playful performativity that bolsters the emphasis on Frank’s age (Fig. 3). The use of the diminutive “Annecke” in the poem as well as in Levi’s apology note also underline her age while performing compassion. Since the modern invention of childhood, children have been imagined and performatively constructed as exceptionally vulnerable and innocent in many contexts (Bernstein 2011; Duschinsky 2013), including Turkey (Altınay 2025). Together with her gender, the innocence associated with childhood informed Frank’s transnational construction as the emblematic Holocaust victim. In fact, adaptations like the 1959 film *The Diary of Anne Frank* (dir. George Stevens, 1959) often temper materials that may affect this image, such as the depictions of her occasionally moody behavior as a teenager or the discussions on her sexuality (Torchin 2012). The poem was thus designed to support the production’s affective politics with its form as well as its content. In the Turkish Jewish context, the acrostic poem exists at an interesting tension. On the one hand, given the use of acrostics in the Hebrew Bible, the poem’s form alludes to a global Jewish identity and culture beyond national borders, grounded in religion. The punctuation after the first letter of each verse, as an effort to ensure that readers understand the form, may simultaneously suggest an alienation from this heritage. On the other hand, the acrostic poem in Turkish functions as a demonstration of linguistic virtuosity. This virtuosity reflects the Jewish struggle to negotiate the politics of national abjection and the impact of the Turkification processes where language played a central role—of which most members of the audience would have a personal memory. In that regard, the unconventional punctuation after the first letters emphasizes Koen’s language skills as well as the labor he put into this ostensibly childlike poem. While serving the Jewish model minority claims, the poem thus became a vehicle for remembering a painful history without violating kayadez.

**Fig. 4:**
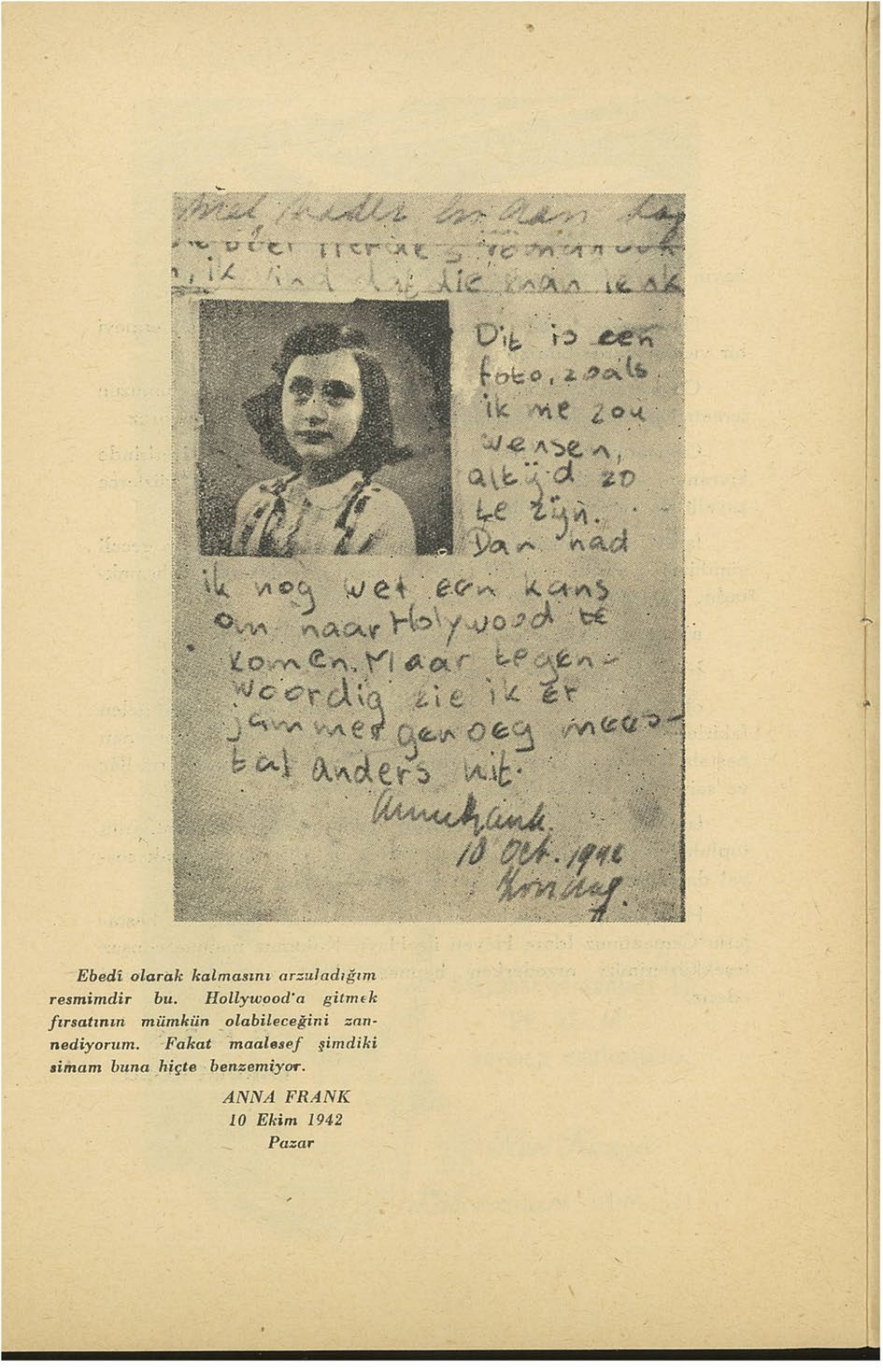
Excerpt from Frank’s diary and her photograph, published with a (mis)translation of the text (G. B. K. Ş. Musevi Cemaati Yardım Kolu 1958, p5)

“Annecke” is not the only acrostic poem in the souvenir book; there is also one about Matan Baseter-Bikur Holim. Founded in 1949 with the merger of two Jewish charity organizations, Matan Baseter-Bikur Holim provided financial support and healthcare services to disadvantaged members of the community. The sentimental poem was signed by the “Charity Branch for Poor Patients/Matan Baseter-Bikur Holim,” but the style suggests that it was probably written by Filon Koen. The two examples of acrostic poetry in the archival volume thus connected the experiences of Turkey’s Jewish community and the globalized narrative of the Holocaust with their form as well as their affective repertoires. This melancholic tone also differentiated the community theatre production from the overly optimistic popular Western adaptations—which, inadvertently, even fed into Holocaust denialism (Cole 1999). The souvenir program book features materials from Frank’s diaries as well. These materials both emphasize the production’s documentary dimension, enhancing its truth-effect, and build connections between Jews in contemporary Turkey and the Holocaust. A striking example of this strategy is found in the first material directly related to the content of the play. On the left, we see a page from Frank’s diary, accompanied by a studio photograph of Frank (Fig. 4). The text reads: “This is a photo, as I wish I could always look like. Then I would still have a chance to come to Hollywood. But these days, unfortunately, I look different most of the time.” The Turkish translation, however, is mostly incorrect: “This is a photograph of mine I wish to last forever. I think it can be possible for me to have the opportunity to go to Hollywood. But unfortunately, my face does not look like this at all at the moment” (G. B. K. Ş. Musevi Cemaati Yardım Kolu 1958, p5). This failure in the translation is also productive. Since Frank (supposedly) desired the photograph to last forever, the producers contributed to the fulfillment of her wish by reproducing it in the archive they created. Moreover, the failed translation’s performative construction of hope, which it attributes to Frank, makes her devastating end even more tragic.

**Fig. 5:**
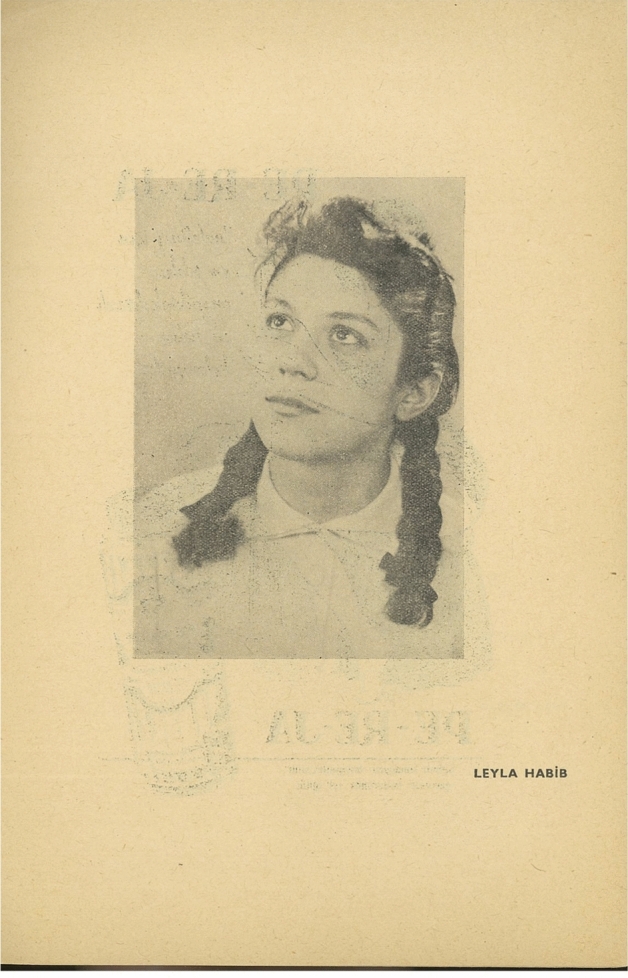
Photograph of Leyla Habib (G. B. K. Ş. Musevi Cemaati Yardım Kolu 1958, p6)

Across from the excerpt from Frank’s diary and her photograph is a studio photograph of the actor playing Frank, Leyla Habib (Fig. 5). In this image, covering the entire page, Habib looks up to her right, like Frank. Art historian Amelia Jones argues that “The photograph, after all, is a death-dealing apparatus in its capacity to fetishize and congeal time” yet “exaggerated theatricality” has the capacity to render photography “eminently performative and so life giving” (Jones 2002, p949). In Leyla Habib’s pose, theatrical performativity has a dual presence at the intersection of everyday and artistic performance. As the actor playing Frank, Habib embodies her, sustaining the object of melancholic attachment and the political possibilities associated with her. Although her dreams of Hollywood stardom are violently crushed by the Holocaust, Anne Frank thus has a new life on the stage and in the archive as a much-beloved and respected protagonist—as also constructed by other materials in this archive of abjection, such as Koen’s poem and Levi’s apology. Moreover, Habib is a Jewish girl herself, with her own dreams and aspirations. The production and the archive suggested that she was able to pursue her dreams thanks to Frank, enhancing the young Holocaust victim’s posthumous power. This put the production in stark contrast with the Ankara State Theatre Production, which did not feature any Jewish actors, and the Hollywood adaptation that would be made the following year, where the non-Jewish actor Millie Perkins was cast as Frank—in part because the persecution of Jewish filmmakers in the context of the Red Scare caused producers to be wary of actors who are “stereotypically” or visibly Jewish as well as productions that are “too Jewish” (Barnouw 2012).

Habib’s performance is a form of surrogation—which, according to the performance theorist Joseph Roach, is “how culture reproduces and re-creates itself” (1996, p2). The performative acts of substitution never result in a perfect sustenance of the order of things; surrogation is always about negotiation and social transformation. “Performance,” in Roach’s framework, “stands in for an elusive entity that it is not but that it must vainly aspire both to embody and to replace” (Roach 1996, p3). As Habib replaces Frank, she does not serve as a perfect substitute. On the one hand, through the performative powers of the theatre and the archive, she maintains Frank’s status as the emblematic Holocaust victim, an “icon of abjection... whose singular experiences come to represent an entire history of abjection through which the cultural borders of a collectivity and its terms of belonging are defined” (Altınay 2024a, p13). Habib thus sustains the melancholia associated with and expressed through Frank in the context of Turkey. On the other hand, even if Frank as a historical figure and play character has died, Habib is very much alive. Her presence on the stage and in the archive activates “the utopian performative,” sustaining the audience’s belief in a better future in the face of trauma, loss, and violence (Dolan 2005). The context of community theatre and associated archival work, functioning as sites of solidarity and community-building, render the utopian politics of performance exceptionally powerful.

As an archive, the souvenir book brings together a diverse array of materials and creates the possibility of imagining and building new connections across them. In the face of kayadez, this unorthodox archive becomes a site for the articulation of historical violence and trauma, countering contemporary antisemitism, and for imagining and investing in the future. Like all archives, the political potentials of *Anne Frank Temsilinin Hatırası* have been reconfigured over the years. With the exploitation of the memory work and archives associated with the Holocaust in the service of Zionism, settler colonialism, and neo-Imperialism, the story of Anne Frank itself has increasingly been deployed in service of violent projects. The imagination of a global Jewish identity and the alternative modes of identification the archive offers thus require us to exercise caution against “the romance of resistance” (Abu-Lughod 1990) that often haunts the literature on minoritarian performances and archives and pay attention to the broader power dynamics at stake.

## Conclusion

Archives serve as a critical venue where abjected minorities explore their pasts and the politics of belonging in the present, develop alternative historiographical narratives, and envision new possibilities. Given theatre’s significance for the negotiations of national abjection, theatre archives offer a particularly promising yet largely neglected site for exploring how these dynamics unfold in the lives of Jews and other post-Ottoman minorities in Turkey. The expanded definition of the archive that has gained prominence with the archival turn can fundamentally advance our understanding of such archives of abjection.

Analyzing Jewish theatre ephemera not simply as archival documents but also as a form of archive in themselves reveals how such materials can provide vital insights into the minoritarian negotiations of belonging in the context of the homogenizing project of the nation-state. The souvenir program book *Anne Frank Temsilinin Hatırası*, designed as an archive, brings together diverse forms of historical and contemporary texts and images. As they contribute to the transmission and transformation of trauma, these materials enable alternative articulations of history, challenging the politics of memory and belonging in the present and enabling new visions and desires for the future. The political interventions involved in such projects, however, may not necessarily be characterized by liberatory or progressive politics. Even more importantly, the meanings of the materials and the political possibilities the archive offers may transform with the reconfiguration of power relations over time. It is with such political promises, ambivalences, and challenges that Turkish Jewish theatre archives await our attention. The absence of well-organized, comprehensive, and accessible collections, however, calls for unorthodox methods—methods that, as this study demonstrates, can yield rich insights into how marginalized communities create meaning through ephemeral archival practices.

## Footnotes

1 All translations from Turkish are by the author unless otherwise noted. This article is part of a project that has received funding from the European Research Council (ERC) under the European Union’s Horizon 2020 Programme for Research and Innovation (Grant Agreement No. 852216 STAGING-ABJECTION). I am grateful to Bilal Akar, Christina Banalopoulou, Angelos Dalachanis, Alexis Rappas, Umut Yamantelli, and the anonymous peer-reviewers for their generous comments and suggestions.

2 The Wealth Tax of 1942 did not target Jews exclusively. Nevertheless, the community was severely affected. Over the years, with the aid of cultural production, including plays such as Aksel Bonfil’s *Varlık* [Wealth] (dir. Aksel Bonfil, 2020), the tax has come to be largely, if not primarily, associated with the Jewish community.
